# Using *in vivo* probabilistic tractography to reveal two segregated dorsal ‘language-cognitive’ pathways in the human brain^☆^

**DOI:** 10.1016/j.bandl.2013.06.005

**Published:** 2013-11

**Authors:** Lauren L. Cloutman, Richard J. Binney, David M. Morris, Geoffrey J.M. Parker, Matthew A. Lambon Ralph

**Affiliations:** a Neuroscience and Aphasia Research Unit (NARU), School of Psychological Sciences, University of Manchester, UK; b Imaging Science and Biomedical Engineering, School of Cancer and Imaging Sciences, University of Manchester, UK

**Keywords:** Arcuate fasciculus, Connectivity, Dual stream model, Functional specialization, Language production, Performance feedback, Repetition, Sensory-motor integration, Supramarginal gyrus, Tool use

## Abstract

Primate studies have recently identified the dorsal stream as constituting multiple dissociable pathways associated with a range of specialized cognitive functions. To elucidate the nature and number of dorsal pathways in the human brain, the current study utilized *in vivo* probabilistic tractography to map the structural connectivity associated with subdivisions of the left supramarginal gyrus (SMG). The left SMG is a prominent region within the dorsal stream, which has recently been parcellated into five structurally-distinct regions which possess a dorsal–ventral (and rostral-caudal) organisation, postulated to reflect areas of functional specialisation. The connectivity patterns reveal a dissociation of the arcuate fasciculus into at least two segregated pathways connecting frontal-parietal-temporal regions. Specifically, the connectivity of the inferior SMG, implicated as an acoustic-motor speech interface, is carried by an inner/ventro-dorsal arc of fibres, whilst the pathways of the posterior superior SMG, implicated in object use and cognitive control, forms a parallel outer/dorso-dorsal crescent.

## Highlights

• The dorsal stream has been postulated to constitute multiple pathways.
• Tractography was used to map the connectivity of regions within the left SMG.
• The arcuate fasciculus was subdivided into dorso-dorsal/ventro-dorsal pathways.
• The parallel pathways appear to underlie functional heterogeneity within the SMG.

## 1 Introduction

Although traditionally conceptualized as a single processing stream, recent evidence from studies of both humans and non-human primates has identified dissociable parallel components in the dorsal pathway, each associated with a different cognitive and language function (Binkofski & Buxbaum, 2012; Catani, Jones, & ffytche, 2005; Catani et al., 2007; Isenberg, Vaden, Saberi, Muftuler, & Hickok, 2012; Kravitz, Saleem, Baker, & Mishkin, 2011; Pisella, Binkofski, Lasek, Toni, & Rossetti, 2006). Within the visuo-motor domain, at least three separate dorsal pathways have been postulated in the non-human primate brain, including a parieto-prefrontal pathway involved in visuospatial processing, a parieto-premotor pathway involved in the visual guidance of action, and a parieto-temporal pathway involved in spatial navigation (Kravitz et al., 2011). Within the human brain, there is also evidence of a division of the dorsal pathway into two subdivisions, one involving the superior parietal lobe, specialised for online actions directed at a visual stimulus based on its structural properties (i.e., reaching and grasping), and one involving the inferior parietal lobe, specialised for actions related to an object’s functional properties (Binkofski & Buxbaum, 2012). However, there is evidence that further dissociations of the dorsal stream, specifically that involving the inferior parietal lobe, may be present, particularly in the linguistic domain (Catani et al., 2005; Catani et al., 2007; Friederici, 2009; Friederici, 2011; Glasser & Rilling, 2008). Studies have found the arcuate fasciculus (AF), a major dorsal language tract, to be composed of two parallel pathways, including a ‘direct’ connection between Broca’s and Wernicke’s areas (corresponding to classical conceptualizations of the AF), and an ‘indirect’ connection between the two regions mediated via the inferior parietal cortex (Catani et al., 2005; Catani et al., 2007). Catani et al. (2005), Catani et al. (2007) postulated that the dissociable AF pathways were associated with separable linguistic functions, with the direct pathway underlying phonological processing and sound-to-motor mapping, and the indirect pathway supporting higher level lexical-semantic language processes.

However, there is some evidence that the anatomical divisions of the AF, specifically the ‘indirect’ frontal-parietal-temporal segment, may be more complex. A dorso-dorsal/ventro-dorsal division of connectivity has been identified in the monkey inferior parietal cortex, with rostral regions connecting via ventro-dorsal pathways and caudal regions via more dorso-dorsal routes (Gregoriou, Borra, Matelli, & Luppino, 2006; Schmahmann & Pandya, 2006). There is some initial evidence that such an organization may also be present in the human brain. A recent study which examined human inferior parietal connectivity via cortico-cortical evoked potentials revealing connections from dorsal and ventral parietal regions to corresponding dorsal and ventral premotor and inferior frontal regions (Matsumoto et al., 2012). This dorso-dorsal/ventro-dorsal organization is also mirrored in functional dissociations, with divisions observed within the left supramarginal gyrus (SMG) associated with cognitive control (dorsal) and phonological encoding-recoding (ventral) (Ravizza, Delgado, Chein, Becker, & Fiez, 2004).

**Fig. 1:**
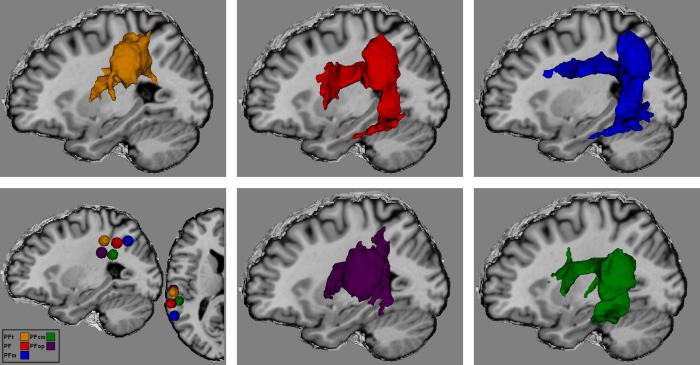
Location of the five left SMG cytoarchitectonic areas used as seed regions for probabilistic tracking (bottom left), depicting the three dorsal SMG regions PFt (orange), PF (red), and PFm (blue), and the two ventral SMG regions PFcm (green) and PFop (purple). Fibre pathways found for each tractographic region are presented. The tracts depicted represent the combined group tracking results (including only those pathways that passed the first-level, i.e., individual subject-level, threshold), transformed into standard MNI space.

The left SMG is a prominent region within the dorsal stream and is an important relay between frontal and temporal brain regions via fibre tracts including the AF (Catani et al., 2005; Catani et al., 2007; Frey, Campbell, Pike, & Petrides, 2008; Parker et al., 2005). Structurally, the SMG has been found to possess a complex cytoarchitecture, and has recently been parcellated using modern techniques into five structurally-distinct regions, roughly organized into a dorsal row of three areas (PFt, PF, PFm) and a ventral group of two entering into the Sylvian fissure (PFop, PFcm; Caspers et al., 2006; Caspers et al., 2008; see Fig. 1). Functional imaging and lesion studies have identified the left SMG to be equally functionally complex, associated with a wide range of cognitive tasks including spatial perception, mental imagery, visuomotor control, motor skill learning and cognitive control (Cabeza & Nyberg, 2000; Nickel & Seitz, 2005; Table 1). An inspection of the functions ascribed to the different cytoarchitectural regions presented in Table 1 reveals a complicated picture, and the mapping between the structural divisions and areas of functional specialisation is by no means one-to-one. Many functional similarities can be indentified across the five SMG sub-regions, however, close examination reveals some potentially informative differences. For example, all subregions appear to be heavily implicated in motor functioning. However, while the dorsal cytoarchitectonic regions are associated with motor planning and execution more generally, the ventral regions appear to be more strongly associated with orofacial movement more specifically.

Both classical and contemporary studies have implicated the left SMG in a variety of language functions, including naming (DeLeon et al., 2007; Pei. et al., 2011), reading (Cloutman, Newhart, Davis, Heidler-Gary, & Hillis, 2011; Hillis et al., 2001), spelling (Cloutman et al., 2009), repetition (Fridriksson et al., 2010), and verbal working memory (Cabeza & Nyberg, 2000). However, the left SMG’s roles in language are unclear. Some studies have suggested a role in multimodal sensory integration and semantic processing (Binder, Desai, Graves, & Conant, 2009), others have implicated the SMG in auditory-motor controlled mappings and phonological processing (Rauschecker & Scott, 2009), while others have argued that the left SMG may be only minimally involved in language, if at all (Glasser & Rilling, 2008). Our working hypothesis for this study was that, rather than being mutually exclusive and rival interpretations of left SMG function, this variation probably reflects the existence of dissociated dorsal pathways between different subregions of the left SMG. However, the underlying neural connectivity of the SMG in humans, and its potential structural–functional subdivisions remain poorly understood.

The current study utilized probabilistic tractography to explore the neural connectivity of the human left SMG, comparing seed regions within the dorsal and ventral SMG. Seed regions for tracking were defined based on the SMG’s underlying cytoarchitecture. The dorsal–ventral nature of the recently defined cytoarchitectural divisions makes these regions useful boundaries in the definition of seed regions for tracking. In addition, both cellular microstructure and neural connectivity are heavily implicated in determining the functional specialization of a region. Cytoarchitecture determines a region’s local processing capabilities whilst its connectivity governs the nature and flow of information to and from an area (Behrens & Johansen-Berg, 2005). Primate studies have observed that functionally and cytoarchitectonically distinct brain regions appear to be associated with distinct cortico-cortical connection patterns, suggesting a strong relationship between brain connectivity and cellular microstructure (Passingham, Stephan, & Kotter, 2002; Rozzi et al., 2006). Importantly, a recent study which utilized tractography to map the underlying anatomical connectivity of the superior (dorsal) SMG (and angular gyrus), found differing patterns of connectivity across the different cytoarchitectural regions explored (Caspers et al., 2011). As such, exploring the connectivity profiles of the five SMG cytoarchitectural regions may help to reveal important differences in its underlying neuroanatomical connectivity, and the existence of separable dorsal stream pathways within the human brain.

## 2 Materials and methods

### 2.1 Participants and image acquisition

Thirteen participants (4 females; mean age = 23.3, range = 19–37) gave written informed consent to participate in the study, which was approved by the local ethics boards. All participants were right-handed, as determined by the Edinburgh Handedness Inventory (Oldfield, 1971).

Imaging data were acquired on a 3T Philips Achieva scanner (Philips Medical Systems, Best, Netherlands), using an 8 element SENSE head coil. Diffusion weighted imaging was performed using a pulsed gradient spin echo echo-planar sequence with TE = 59 ms, TR ≈ 11884 ms (cardiac gated), *G* = 62 mT m^−1^, half scan factor = 0.679, 112 × 112 image matrix reconstructed to 128 × 128 using zero padding, reconstructed resolution 1.875 × 1.875 mm, slice thickness 2.1 mm, 60 contiguous slices, 61 non-collinear diffusion sensitization directions at *b* = 1200 s mm^−2^ (*Δ* = 29.8 ms, *δ* = 13.1 ms), 1 at *b* = 0, SENSE acceleration factor = 2.5. Each diffusion weighted volume was acquired entirely before starting on the next diffusion weighting, resulting in 62 temporally spaced volumes with different diffusion gradient directions. For each diffusion gradient direction, two separate volumes were obtained with opposite directional *k*-space traversal (and thus reversed phase and frequency encode direction), with phase encoding in the left–right/right–left direction in order to reduce signal distortion (Embleton, Haroon, Morris, Lambon Ralph, & Parker, 2010). Acquisitions were cardiac gated using a peripheral pulse unit positioned over the participant’s index finger (*n* = 10), or an electrocardiograph (*n* = 3). The diffusion weighted images were corrected for susceptibility- and eddy current-induced distortion using the method described in Embleton et al. (2010). A co-localized T_2_-weighted turbo spin echo scan, with in-plane resolution of 0.94 × 0.94 mm and slice thickness 2.1 mm, was obtained as a structural reference scan to provide a qualitative indication of distortion correction accuracy. A high resolution T_1_-weighted 3D turbo field echo inversion recovery scan (TR ≈ 2000 ms, TE = 3.9 ms, TI = 1150 ms, flip angle 8°, 256 × 205 matrix reconstructed to 256 × 256, reconstructed resolution 0.938 × 0.938 mm, slice thickness 0.9 mm, 160 slices, SENSE factor = 2.5), was also acquired for the purpose of high-precision anatomical localization of seed regions for tracking.

### 2.2 Definition of regions of interest

Regions of interest (ROIs) for white matter tractography were defined for the five left hemisphere SMG cytoarchitectonic regions identified by recent parcellations (Caspers et al., 2006; Caspers et al., 2008). For each cytoarchitectonic region, a 5 mm spherical ROI (515 voxels) was drawn onto the ICBM single-subject brain template (in anatomical MNI space; International Consortium for Brain Mapping, http://www.loni.ucla.edu/ICBM/Downloads/Downloads_ICBMtemplate.shtml), with the centre of the sphere positioned at the anatomical MNI co-ordinates of the region’s centre of gravity, as reported by Caspers et al. (2008; Fig. 1A). Probabilistic cytoarchitectonic maps of the left SMG regions from the SPM Anatomy toolbox (Eickhoff et al., 2005), were used as masks to (a) ensure that no voxel in the ROI was outside a region’s probabilistic anatomical boundaries, and (b) ensure that the seed ROIs optimally included predominantly the SMG cortex and a small amount of underlying gyral white matter, minimizing the chance that the ROI included white matter tracts that did not connect with the cortical surface. For fibre tracking, the ROIs were transformed from anatomical MNI space into each participant’s native diffusion space, using the DARTEL toolbox supplied as part of SPM8 (Statistical Parametric Mapping; http://www.fil.ion.ucl.ac.uk/spm; Ashburner, 2007). The high-resolution T1-weighted images, linearly co-registered with the diffusion weighted images, were used for the registration and to confirm the accuracy of the transformation of the ROIs into native space.

### 2.3 Fibre tracking and anatomical localization of fibre pathways

Unconstrained probabilistic tractography was performed with a dedicated software package using the PICo method (Parker, Haroon, & Wheeler-Kingshott, 2003). This method utilizes a Monte Carlo approach for streamline propagation, sampling the orientation of probability distribution functions (PDFs, generated based on uncertainty in eigenvector orientation, using the constrained spherical deconvolution method; Tournier, Calamante, & Connelly, 2007; Tournier et al., 2008), within each voxel, and advancing the streamline in the direction of the interpolated modified principal eigenvector. The streamline tracking process is repeated multiple times, with the number of streamlines which encounter each voxel within the brain recorded, allowing for the calculation of the maximum connectivity from voxels in the start region to a given voxel in the brain. In the current study, 10,000 streamlines were initiated from each voxel within an ROI. Step size was set to 0.50 mm. Stopping criteria for the streamlines were set so that tracking terminated if pathway curvature over a voxel was greater than 180°, or the streamline reached a physical path limit of 500 mm.

The cortical brain regions associated with each fibre pathway were determined using brain region masks from the AAL atlas, generated using the WFU Pick Atlas (Maldjian, Laurienti, Burdette, & Kraft, 2003; Tzourio-Mazoyer et al., 2002). Due to the large size of the brain masks, and interest in potential functional differences between identified sub-regions within these areas, the AAL masks for the insula and temporal gyri were divided into anatomical sub-regions: the insula was divided into anterior and posterior subdivisions based on recent anatomical descriptions (Naidich et al., 2004); the superior, middle, and inferior temporal gyral masks were subdivided into rostral and caudal subdivisions, which were defined by a vertical division lying perpendicular to the anterior commissure–posterior commissure (AC–PC) plane, which bisected the brain at approximately the midway point between the AC and PC. This resulted in 49 target regions covering the whole of the left hemisphere (excluding the SMG).

To allow for anatomical localization and inter-subject comparisons, the tracking results for each participant were spatially normalized into a common space. The DARTEL registration involves two transformation matrices when registration is performed between native and standard space: a nonlinear deformation matrix between each participant’s diffusion space and a group average template space, and a common linear matrix between the group template space and the MNI template space. In the current study, the group template space was chosen as the common space as this required only one interpolation for each transformation (i.e., individual-to-group and MNI-to-group), reducing the potential for interpolation artefacts.

For each cytoarchitectonic region, the AAL masks were overlaid over each participant’s spatially transformed tracking data to obtain a maximum connectivity value (ranging from 0 to 10,000), between each cytoarchitectonic seed region and all areas of the brain. The resultant streamline-based connectivity matrices were subjected to a double threshold to ensure that only connections with a *high probability* in the *majority* of participants were considered (Cloutman, Binney, Drakesmith, Parker, & Lambon Ralph, 2012). At the first-level, an individual threshold was statistically established as follows. For each participant, the maximum connectivity values for each hemisphere across all ROIs and AAL brain regions were used to determine the distribution of connection values for that hemisphere between the SMG and all other (ipsilateral) areas of the brain. The *λ*-value of the Poisson distribution identified was then used to determine a threshold value at *p* = .05, above which a connection between an ROI and brain region was deemed to exist with a high degree of probability. At the second-level group stage, from the set of individual high-probability connections, only those that were consistently identified across participants were selected, using both a stringent (over 75% of participants, i.e., at least 10/13 participants) and a more relaxed (over 50% of participants, i.e., at least 7/13 participants) criteria for consistency.

## 3 Results

**Fig. 2:**
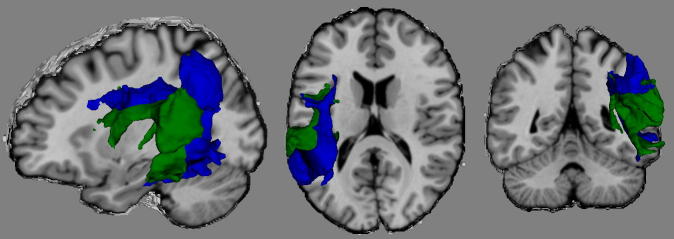
Comparison of a representative dorso-dorsal pathway (blue, PFm) and a ventro-dorsal pathway (green, PFcm). The fibre pathways depicted represent the combined group tracking results transformed into standard MNI space.

The connectivity profiles for each SMG ROI are presented in Table 2, with the associated fibre tracts (spatially normalized into MNI template space and combined across the group) projected onto the brain in Figs. 1 and 2.

Examination of the left SMG connectivity profiles reveals different patterns of connectivity across the various seed regions, each associated with dissociated dorsal pathways. The inferior SMG (combining PFop and PFcm) showed connectivity dorso-rostrally to the inferior frontal, motor and somatosensory areas, and the insula, and ventrally to multiple anterior and posterior temporal regions, with additional connectivity for PFop with the primary auditory cortex. Posterior superior SMG regions PF and PFm demonstrated a different pattern of connectivity to that of the inferior SMG regions, with dominant connectivity to motor and posterior temporal regions, and an absence of connectivity with the insula or anterior temporal areas. While PF and PFm were both found to connect to frontal regions, there was some dissociation between them, with PF connecting to inferior frontal regions, while PFm was connected with the middle frontal gyrus. These contrastive connectivity patterns reflect a dissociation of fibre bundles within the AF itself (see Fig. 2). Specifically, the connectivity of the inferior SMG is carried by an inner/ventral arc of fibres, whilst the pattern for the posterior superior SMG (specifically region PFm) forms a parallel, outer/dorsal crescent. For region PF, the vertical portion of the AF demonstrated strong overlap with PFm, while along the horizontal portion of the AF, the fibre pathways demonstrated overlap with those of both PFm and the inferior SMG. Unlike the superior SMG regions, the inferior SMG regions also demonstrated additional neural connections via a tract consistent with the extreme capsule (Makris & Pandya, 2009). Finally, the anterior superior region (PFt) demonstrated a very selective pattern of connections to motor and somatosensory areas via relatively short fibre tracts, potentially involving a portion of SLF III, with the associated fibre tracts located more laterally than compared to those found for PF and PFm (Fig. 1; Makris et al., 2005).

## 4 Discussion

Examination of the SMG connectivity profiles and associated fibre pathways within the current study revealed three major left SMG subdivisions: (1) an inferior SMG region (involving PFop and PFcm), connected to frontal and temporal regions via an inner ventro-dorsal crescent of the AF and the extreme capsule; (2) a posterior superior SMG region (involving PF and PFm), connected via an outer dorso-dorsal AF crescent; and (3) an anterior superior SMG region (involving PFt), selectively connected to pre- and postcentral gyri via relatively short fibre tracts, likely including SLFIII. Due to the invasive nature of traditional techniques for studying connectional architecture, previous studies of the neutral connectivity of the SMG have predominantly involved primate models. These studies have observed patterns of inferior parietal cortex (including the SMG homologue) interconnection with a widely distributed network of brain regions similar to that found in the current study, including auditory areas in the posterior temporal lobe, somatosensory regions, and frontal regions including the precentral, inferior frontal, and middle frontal gyri (Andersen, Asanuma, Essick, & Siegel, 1990; Pandya & Seltzer, 1982; Rozzi et al., 2006; Seltzer & Pandya, 1984). The connectivity profile of the left SMG found in the current study also corresponds well with the small number of previous studies of the structural and functional connectivity of the human inferior parietal cortex, which also observed connectivity between the human SMG and regions including the middle and inferior frontal gyri, posterior temporal regions, and the insula (Caspers et al., 2011; Nelson et al., 2010; Uddin et al., 2010). The dominance of the AF observed in the current study is highly consistent with the tracts identified by Caspers et al. (2011), who also examined the connectivity of the dorsal SMG cytoarchitectonic regions. However, there were some notable differences, particularly in relation to region PFt, which was found to have far greater connectivity (via the AF), than was observed in the current study. One possible explanation for the discrepancy may be due to methodological differences between the two studies in the determination of tract probability thresholding. However, another key difference between this and the previous study is in relation to the seed ROIs used. The study of Caspers et al. (2011) defined their ROIs via maximum probability maps, and tracking was performed across an entire cytoarchitectonic region rather than a small area within its centre, as was done in the current study. The use of the maximum probability maps may have resulted in the ROIs covering more than one cytoarchitectonic region (many individual voxels have a probability of belonging to two or more different regions), and the tracking from transitional zones which could have potentially produced patterns of hybrid connectivity for some proximal regions.

Previous studies with both humans and primates have identified the dorsal stream as constituting multiple dissociable parallel pathways associated with a range of specialized cognitive functions (Binkofski & Buxbaum, 2012; Catani et al., 2005; Catani et al., 2007; Kravitz et al., 2011). Within the human brain, researchers have previously postulated a dorso-dorsal and ventro-dorsal subdivision (Binkofski & Buxbaum, 2012). However, in contrast to the current study, the ventro-dorsal pathway has been associated with the inferior parietal lobe (including the SMG), while the dorso-dorsal route has been associated with a pathway involving the *superior* parietal lobe. Thus, the current study differs from previous conceptualisations of dorsal stream subdivisions by further bifurcating the inferior parietal pathway into dorso-(ventro-)dorsal and ventro-(ventro-)dorsal subdivisions.

Importantly, this dorso-dorsal/ventro-dorsal division of connectivity in the inferior parietal cortex has been identified in several previous human and primate studies, with rostral regions connecting via ventro-dorsal pathways and caudal regions via more dorso-dorsal routes (Gregoriou et al., 2006; Matsumoto et al., 2012; Schmahmann & Pandya, 2006). This same pattern of dissociable dorso-dorsal/ventro-dorsal connectivity was observed in the current study, with inner/ventral and parallel outer/dorsal AF crescents. Interestingly, the current inner and outer AF pathways identified are almost identical to that observed in a recent study which used probabilistic tractography to map the connective pathways of the anterior and posterior planum temporale (Isenberg et al., 2012). Thus, there is increasing evidence that the ‘indirect’ human dorsal AF pathway identified by Catani et al. (2005), Catani et al. (2007) may involve more complex and fine-grained divisions. It seems likely that the functional heterogeneity ascribed to regions of the left SMG in previous functional imaging studies may be a direct reflection of the parallel yet dissociated pathways found in this study. Possible functions of the segregated pathways will briefly be discussed.

Studies in both humans and primates have implicated a key functional role for the SMG in the transformation of sensory input into motor output, and the sensory guidance of behaviour (Binkofski & Buxbaum, 2012; Della-Maggiore, Malfait, Ostry, & Paus, 2004; Desmurget & Grafton, 2000; Desmurget et al., 2009; Rozzi, Ferrari, Bonini, Rizzolatti, & Fogassi, 2008; Rozzi et al., 2006). Such a processing role would be heavily involved in a range of cognitive skills previously ascribed to the SMG including language production, the orienting of attention in response to external stimuli, hand-object interactions, and action observation and imitation ( Demonet, Thierry, & Cardebat,2005; Price, 2010; Ptak, 2012; Ramayya, Glasser, & Rilling, 2010; Rozzi et al., 2006). Additionally, this would also implicate an important role of the SMG in the monitoring and adjustment of performance through sensory-motor feedback loops, necessary for the acquisition and execution of skilled movement (Guenther, 2006; Rauschecker & Scott, 2009). The current tractography results appear to support this sensory-motor functional hypothesis, and a consistency in connectivity was observed across the SMG with brain regions involved in sensory (auditory) and somatosensory input and processing, and fine-motor co-ordination and planning. Thus, a set of core processing capabilities and a commonality of function appear to be at the heart of the structural and functional organization of the SMG. However, the structural/functional subdivisions and dissociated fibre pathways observed in this and previous studiesindicate a strong degree of functional segregation within the SMG, and regions with functionally specialized roles within this sensory-motor network.

The SMG has been found to be associated with orofacial movements in both humans and primates (Buccino et al., 2001; Desmurget et al., 2009; Rozzi et al., 2008). However, a degree of functional lateralization has been observed in the human inferior SMG, particularly in cytoarchitectonic region PFcm, with stimulation of this area in the right hemisphere resulting in an intent to move the hand, arm, or foot, while left hemisphere stimulation provoked a intention to move the lips and speak (Desmurget et al., 2009). In the current study, the left inferior SMG (PFcm, PFop) connected with a number of brain regions heavily implicated in language, including posterior temporal areas (including the auditory cortex on Heschl’s gyrus), Broca’s area, and the primary motor cortex on the precentral gyrus. Studies of both primates and humans have identified theseregions within a network associated with the translation of auditory information into motor action, important for the learning of novel actions through mimicry (Hamzei et al., 2003; Petrides & Pandya, 2009). Consistent with this, it has been suggested that the auditory-motor translation network has been adapted in the left hemisphere of humans for sound-to-speech transformations (Rauschecker & Scott, 2009), ultimately allowing repetition of novel, meaningless words and sentences without reference to meaning (with damage to this network producing various forms of conduction aphasia: Hickok & Poeppel, 2004). Within this dorsal stream ‘repetition-phonological’ network, previous studies have implicated a role for posterior temporal regions in the transient representation of the phonetic sound sequences to berepeated (Scott, Blank, Rosen, & Wise, 2000; Wise et al., 2001), while frontal regions including Broca’s area, the insula and the motor cortex have been associated with the translation of this phonetic information into vocal tract motor patterns for articulation (Baldo, Wilkins, Ogar, Willock, & Dronkers, 2010; Eickhoff, Heim, Zilles, & Amunts, 2009; Price, 2010). The precise role of the inferior SMG region within this dorsal repetition network remains unclear, however, an increasing number of studies have identified this area as a key region associated with phonological processing (Hartwigsen et al., 2010; Kircher, Nagels, Kirner-Veselinovic, & Krach, 2011; Maldonado, Moritz-Gasser, & Duffau, 2011; Maldonado et al., 2011; Prabhakaran, Blumstein, Myers, Hutchison,& Britton, 2006; Ravizza et al., 2004; Salmelin & Kujala, 2006). Specifically, the SMG has been implicated in phonological working memory, a system heavily involved in successful repetition (Dien, 2009; Jonides et al., 1998; Vigneau et al., 2006), and recent functional imaging studies have implicated the SMG in the processing of syllable order of auditory speech sounds (Moser, Baker, Sanchez, Rorden, & Fridriksson, 2009). In addition, there is increasing evidence for the importance of the SMG in feedback processes associated with the coordination of articulatory movements for speech production and speech motor learning (Golfinopoulos et al., 2011; Shum, Shiller, Baum, & Gracco, 2011). All of these language-related functions follow from the more general processing assumption that inferior SMG (and inferior parietal cortex more generally) may act as a key feed-forward and feedback auditory-motor interface (Rauschecker, 2011; Rauschecker & Scott, 2009), and the current *in vivo* tractography data support this more general hypothesis (see also Parker et al., 2005) as do recent neuroanatomically-constrained models of spoken language (Ueno, Saito, Rogers, & Lambon Ralph 2011).

The posterior superior SMG regions (PF/PFm) formed a dorso-dorsal network with posterior temporal, pre- and postcentral, and frontal areas – regions associated with semantic, motor, and somatosensory processing (Cabeza & Nyberg, 2000). As noted in the Introduction, the left SMG has been associated with a wide range of cognitive skills including mental imagery, motor-skill learning and spatial processing. This posterior superior SMG region has been particularly implicated in the cognitive processes involved in hand-object interactions and tool use (Binkofski & Buxbaum, 2012; Goldenberg & Spatt, 2009; Grafton & Arbib, 1996; Nickel & Seitz, 2005; Randerath, Goldenberg, Spijkers, Li, & Hermsdorfer, 2010; Saccuman et al., 2006). In addition, previous studies have suggested that action knowledge may be represented in the SMG, where abstract somatosensory knowledge acquired during the learning of skilled motor sequences may be stored (Binder & Desai, 2011; Binder et al., 2009; Mahon, Schwarzbach, & Caramazza, 2010). The pattern of more limited connectivity found for the anterior superior SMG region (PFt), is consistent with its function also falling into this general processing domain. More specifically, this region may play a selective role in the translation/integration of somatosensory and motor information, whilst the posterior superior SMG area may integrate a much wider range of semantic and somatosensory information, required for successful object use (Glasser & Rilling, 2008; Ramayya et al., 2010). These current dorsal SMG pathways would map closely to the previously identified human dorsal route subdivision involving the inferior parietal lobe, which was postulated to be involved in sensorimotor processing based on semantic object use information, specialised for function-based object-related actions (Binkofski & Buxbaum, 2012).

### 4.1 Methodological considerations and limitations of the current study

Since it was discovered that the diffusion direction of water molecules could be used to infer the orientation and course of white matter fibre tracts in the brain *in vivo*, a plethora of studies have been conducted using the diffusion tractography technique. Importantly, in tractography, the presence, absence and direction of neural fibre pathways is not visualized directly but must be indirectly inferred, and there are multiple sources of error which may affect the validity of the fibre pathways identified. One of the principal sources of error are modelling errors during tract reconstruction resulting from factors such as partial volume effects, the branching of fibre pathways and the length and shape of the paths tracked. As such, there is a level of uncertainty in all tractographic data, with a degree of both false positives (Type I errors) and false negatives (Type II errors) inherent in any set of results. A key focus of recent research has been the methodological refinement of the tractographic technique to address these issues, and substantial advances have been made in relation to the modelling of complex tissue fibre orientations, and the way in which the uncertainty in fibre orientation is derived and sampled (e.g., Behrens, Johansen-Berg, Jbabdi, Rushworth, & Woolrich, 2007; Chung, Lu, & Henry, 2006; Haroon, Morris, Embleton, Alexander, & Parker, 2009; Jian & Vemuri, 2007; Jones & Pierpaoli, 2005; Lazar & Alexander, 2005). Within the current study, a sophisticated combination of probabilistic tractography using the PICo method (Parker et al., 2003), and constrained spherical deconvolution (CSD; Tournier et al., 2007; Tournier et al., 2008), was implemented to benefit from these recent advancements and increase the anatomical accuracy and validity of the pathways identified.

Traditional tracking techniques utilised the deterministic tractography approach, in which a single streamline is propagated bi-directionally from a seed region along the line of the principle eigenvector of the diffusion tensor (e.g., Basser, Pajevic, Pierpaoli, Duda, & Aldroubi, 2000; Conturo et al., 1999; Mori, Crain, Chacko, & Van Zijl, 1999). However, as there is only one reconstructed trajectory per seed region, deterministic methods are associated with two important limitations: (1) they are unable to provide a measure of the uncertainty of the reconstructed pathways (Jones, 2008; Jones, 2010); (2) in regions of complex fibre architecture (specifically, voxels containing more than one fibre orientation, such as crossing/kissing fibres, or the branching of fibre pathways), the tensor model is inadequate as there is only one estimate of fibre orientation and reconstructed trajectory per voxel, a significant issue given the estimation that as many as 90% of voxels may contain such complex orientations (Jeurissen, Leemans, Tournier, Jones, & Sijbers, 2012). In contrast, probabilistic tractography techniques, such as the PICo method used in the current study, are able to overcome these limitations by taking into account the local uncertainty in fibre orientation and repeating streamline propagation multiple times, allowing for an estimation of probability for any pathway reconstructed (Behrens et al., 2003; Parker et al., 2003).

Instead of a simple diffusion tensor, probabilistic algorithms repeatedly sample probability distribution functions (PDFs) that describe the uncertainty of local fibre orientation distributions. In the current study, PDFs were generated using the constrained spherical deconvolution method (Tournier et al., 2007; Tournier et al., 2008). This technique provides an estimate of the distribution of possible fibre orientations within a given voxel by assuming that all white matter fibre populations share identical diffusion characteristics and may be described by a common signal profile (the response function). As a consequence, any differences in anisotropy may be attributed to partial volume effects (see Tournier, Calamante, Gadian, & Connelly, 2004; Tournier et al., 2007, for full details of the method). The product of the CSD is a spherical function which provides information on the number and direction of the orientations present within a given voxel, as well as their relative weightings, referred to as the fibre orientation distribution (FOD). This FOD may be sampled via techniques such as model-based residual bootstrapping to obtain an estimate of the uncertainty in fibre orientations produced by the CSD analysis and generate the PDF (Chung et al., 2006; Haroon et al., 2009).

The probabilistic tractography and CSD methods were implemented in the current study due to substantial evidence regarding their efficacy and superiority over other methods, notably in relation to their ability to resolve narrow crossing fibre angles (as small as 30°; Tournier et al., 2008), and produce robust and reproducible tracking results which correspond well to known anatomy (Jeurissen, Leemans, Jones, Tournier, & Sijbers, 2011; Ramirez-Manzanares, Cook, Hall, Ashtari, & Gee, 2011; Tournier, Calamante, & Connelly, 2012). Indeed, these methodologies are quickly becoming the most widely used techniques, and have been implemented in a number of recently developed tractography tools (e.g., MRtrix; Tournier et al., 2012). There is an increasing body of evidence regarding the accuracy of the white matter fibre pathways delineated by the methods in combination, and studies have identified fibre tracts which correspond well to those identified via primate tracer and human anatomical dissection studies (e.g., Catani et al., 2012; Reijmer et al., 2012; Thiebaut de Schotten, Dell’Acqua, Valabregue, & Catani, 2012). In addition, the specific combination of PICo/CSD employed in the current study has been used in previous studies by our research group to successfully delineated the neural connectivity of brain regions including the insula (Cloutman et al., 2012), and the anterior temporal lobe (Binney, Parker, & Lambon Ralph, 2012). However, it is important to acknowledge that the tractography methods used in this and previous studies are new and innovative techniques which continue to require further exploration, evaluation and validation, a task beyond the scope of the current paper.

Despite the substantial advancements which continue to be made to the tractographic technique, important limitations still remain which need to be acknowledged and considered when interpreting the results of any tractography study (for reviews, see Jbabdi & Johansen-Berg, 2011; Jones 2008; Jones 2010). The key limitations of relevance to the current study are associated with the problems of distance effects and thresholding (Jones, 2008; Morris, Embleton, & Parker, 2008). In probabilistic tractography, the propagation of streamlines is repeated multiple times (usually in the order of ‘000s), with the number of times each voxel is reached by the advancing streamlines allowing for an estimation of connection probability and a measure of the confidence which can be assigned to an identified route. However, the advancement of the tractographic streamline is associated with an accumulation of uncertainty, due to the uncertainty in fibre orientation within each voxel discussed above. The product of this propagation of uncertainty is a decrease in connection probability with increasing path length, leading to a preponderance of high probability connections close to the seed region coupled with a progressive dispersion of low probability streamlines as distance increases (Morris et al., 2008). Consequently, this results in increased difficulty in tracking long-range connections, as well as in the interpretation of any tracking values, as the probability of connection is not uniform across distance. As such, it is difficult to determine a threshold value which will successfully identify true positives while simultaneously minimising the rate of both Type I errors in regions close to the seed and Type II errors in more distant regions. In addition, while studies have begun to develop statistical techniques to utilise quantitative streamline density values in the analysis of tractographic output (e.g., Caspers et al., 2011; Iturria-Medina et al., 2007), these values are difficult to interpret in any absolute way (Bastiani, Shah, Goebel, & Roebroeck, 2012; Jbabdi & Johansen-Berg, 2011; Jones, 2008; Jones, 2010). Due to the current lack of clarity regarding how these quantitative values should be utilised, the current study took a conservative approach of utilising these values for thresholding only. Streamline density was used to define a threshold value by taking the average of the IPL connectivity distribution across the entire brain, reflecting values from regions with both short and long connectivity distances. This most likely resulted in a conservative cut-off value for longer pathways, and it is important to acknowledge that there may be long-range connections left undetected in the current study. However, it is believed that the high cut-off value used would likely have produced fewer false positives in the long range connections and fibre pathways identified.

The current study delineated two subdivisions of the AF based on the anatomical dissociation of the pathways in relation to their course and differences in termination patterns. However, this is not to suggest that these are the only subdivisions present within the AF. Indeed, with the use of a greater number of smaller and more fine-grained targets, additional dissociations of further functionally specialised pathways may be identified. However, it is important to note that target region size and number are not the principal determinants in identifying different functional pathways. Studies which have attempted to parcellate the cortex into structurally and functionally coherent regions have found that the use of a small number of comparatively large cortical target regions (such as was used in the current study) can define structural/functional subdivisions highly similar to that obtained by studies which utilised voxel-sized targets (Traynor et al., 2010). The use of large, anatomically-defined cortical regions and target area analyses in the examination of a given brain region’s connectivity profile benefits over more voxel-based analyses as it enables potential functional roles to be more easily inferred. In addition, the use of large cortical target regions has been argued to produce more reproducible tractography results, and reduce inter-subject variability (Traynor et al., 2010).

## 5 Conclusions

The current study provides additional support for the existence of multiple dissociable parallel dorsal stream pathways, and extends previous findings to further subdivide the ‘indirect’ frontal-parietal-temporal AF pathway into dorso-dorsal and ventro-dorsal tracts. Previous functional imaging and lesion studies have identified the left SMG as a functionally complex region implicated in a wide range of cognitive skills, and it is postulated that the functional heterogeneity ascribed to regions of the left SMG may be a direct reflection of the parallel pathways found in this study. Importantly, there is a parallelism between the functions ascribed to the inferior versus superior SMG and the underlying neural pathways identified. Both regions are assumed to play a key role in sensory-motor mapping and feedback, arising from similar networks of connectivity with motor, somatosensory, inferior frontal, and temporal brain areas. The division between the auditory-oral motor (inferior SMG) versus visuosemantic-hand motor (superior SMG) functions appears driven (at least in part) by the segregation of the underlying connective pathways involved, with the inner/ventro-dorsal AF providing the auditory-motor connectivity for the inferior SMG, whilst a parallel outer/dorso-dorsal AF crescent supports the connectivity for the superior SMG. If this is correct then it provides a clear example for the power of neural connectivity on cortical function.

## Figures and Tables

**Table 1:** Cognitive functions associated with the five SMG cytoarchitectonic regions.

	Motor function	Cognitive control	Language
PF	Motor sequencing and sequential movement (Bischoff-Grethe, Goedert, Willingham, & Grafton, 2004; Mallol et al., 2007)	Response switching (Rushworth, Paus, & Sipila, 2001)	Action/manipulable object naming (Berlingeri et al., 2008; Saccuman et al., 2006)
	Object manipulation (Creem-Regehr & Lee, 2005)	Response inhibition (Chikazoe, Jimura, Hirose, et al., 2009; Kelly et al., 2004)	Syllable/sentence sequencing (Bohland & Guenther, 2006; Peck et al., 2004)
	Motor planning (Hanakawa, Dimyan, & Hallett, 2008; Johnson-Frey, Newman-Norlund, & Grafton, 2005)	Performance monitoring (Tang, Critchley, Glaser, Dolan, & Butterworth, 2006)	
	Execution of movement (Filimon, Nelson, Hagler, & Sereno, 2007; Kuhtz-Buschbeck et al., 2003)		
	Action recognition (Hamzei et al., 2003)		

PFm	Execution of movement (Kroliczak, Cavina-Pratesi, Goodman, & Culham, 2007)	Performance monitoring (Ullsperger & von Cramon, 2001)	Action/manipulable object naming (Liljestrom et al., 2008; Rowan et al., 2004; Vitali et al., 2005; Warburton et al., 1996)
		Working memory (Carlson et al., 1998)	Sentence construction (Kemeny, Ye, Birn, & Braun, 2005)
			Lexical decision (Binder et al., 2003)

PFt	Motor sequencing and sequential movement (Rektor, Rektorova, Mikl, Brazdil, & Krupa, 2006; Samuel et al., 1997)	Response inhibition (Chikazoe, Jimura, Asari, et al., 2009)	Syllable sequencing (Bohland & Guenther, 2006)
	Imagined/observed movement (Gerardin et al., 2000; Grafton & Arbib, 1996; Harrington, Farias, Davis, & Buonocore, 2007)		Syllable production (Thompson et al., 2007)
			Object naming (Saccuman et al., 2006)

PFcm	Motor sequencing and sequential movement (Mallol et al., 2007)	Response inhibition (Liu, Banich, Jacobson, & Tanabe, 2004; Peterson et al., 2002)	Phonological processing (Xu et al., 2002)
	Orofacial imitation (Calvert & Campbell, 2003; Lee, Josephs, Dolan, & Critchley, 2006)		Syntactic processing (Luke, Liu, Wai, Wan, & Tan, 2002)
	Orofacial somatosensation/movement (Lowell et al., 2008)		

PFop	Motor sequencing and sequential movement (Daselaar, Rombouts, Veltman, Raaijmakers, & Jonker, 2003; Samuel et al., 1997)	Response conflict (Barch et al., 2001)	Syllable production (Ghosh, Tourville, & Guenther, 2008)
	Object manipulation (Creem-Regehr & Lee, 2005)		Action naming (Damasio et al., 2001)
	Motor planning (Johnson-Frey et al., 2005)		Synchronisation of singing (Saito, Ishii, Yagi, Tatsumi, & Mizusawa, 2006)
	Execution of movement (Joliot et al., 1999)		
	Orofacial imitation (Leslie, Johnson-Frey, & Grafton, 2004)		
	Orofacial somatosensation/movement (Lowell et al., 2008; Martin et al., 2004)		

**Table 2:** Connectivity profiles (as measured by number of participants above threshold) for each left SMG region.

NB: Numbers in bold represent a strict consistency criteria of over 75% of participants (i.e., at least 10/13 participants), while numbers not in bold represent a relaxed consistency criteria of over 50% of participants (i.e., at least 7/13 participants). Only those target regions which displayed a significant connection with the SMG are presented.
